# Spatial Distribution and Potential Health Risks of Arsenic (As) and Associated Metals (Fe and Mn) in the Coastal Accreted Land of Meghna River Estuary and Their Implication on the Agricultural Aspects

**DOI:** 10.1155/sci5/8891363

**Published:** 2025-03-12

**Authors:** Tabarok Bhuiyan, Fahmida Akter, Yeasmin Nahar Jolly, Md. Jamiul Kabir, Mohammad Abdul Momin Siddique

**Affiliations:** ^1^Department of Oceanography, Noakhali Science and Technology University, Noakhali 3814, Bangladesh; ^2^Atmospheric and Environmental Chemistry Laboratory, Chemistry Division, Atomic Energy Centre, Dhaka 1000, Bangladesh; ^3^Faculty of Fisheries and Protection of Waters, South Bohemian Research Center of Aquaculture and Biodiversity of Hydrocenoses, Research Institute of Fish Culture and Hydrobiology, University of South Bohemia in Ceske Budejovice, Zatisi 728/II, Vodnany 389 25, Czech Republic

**Keywords:** coastal agriculture, Meghna River estuary, metal pollution, potentially toxic elements, soil quality

## Abstract

Arsenic (As),iron (Fe), and manganese (Mn) pollution in the coastal areas of Bangladesh are severe problems.*Irrigation* by shallow wells in the agricultural lands is the primary source of these metals. Being a part of the Ganges, Brahmaputra, and Meghna (GBM) Delta, the coastal accreted land of the Meghna River estuary has experienced a series of erosion and accretion phenomena and deposited a vast amount of sediments along with potentially toxic elements. This study investigated the spatial distribution, source, fate, and potential environmental and human health risks of As, Fe, and Mn from 25 sites across the coastal accreted land in the lower Meghna River estuary, Bay of Bengal. The mean concentration of As, Fe, and Mn in the surface soil samples ranged from 0.1–5.16, 12,000–23,810, and 50.6–1025.12 mg/kg, respectively, where high concentrations of metals were found in the southern belt of the estuary. A high As concentration (> 2 mg/kg) was observed at stations 3-4, 15, and 17. Igeo values of As, Fe, and Mn were estimated as −1.05, −0.50, and −0.55, respectively. The Igeo values analyzed in the sediments were below zero for all the metals, suggesting no contamination from these metals. The pollution load index (PLI) for As, Fe, and Mn was lower than the contamination level, indicating that contamination levels remain below harmful thresholds but require regular monitoring. Potential ecological risk index (PERI) values (1.32–10.75) showed low ecological risks in the studied area. Moreover, “no risk” to “low level” of carcinogenic risk was identified. According to the threshold values, except in the southern belt (stations 3-4, 15, and 17), most of the accreted agricultural land can be considered adequately safe for food production. This study suggests that plant analyses be incorporated into future research; however, it would be more impactful to emphasize bioavailability studies and their relevance to agricultural safety.

## 1. Introduction

Arsenic (As) is a metalloid and, as a carcinogen agent, does not benefit living organisms, even at low levels [1]. Arsenic is characterized by its facile alteration in oxidation state and bonding configurations with associated metals, including iron (Fe) and manganese (Mn). Arsenic exhibits versatile chemical behaviors within the environment and forms numerous organic and inorganic compounds [2]. Iron and Mn oxides act like binders for As in rock formations. The specific bonding patterns are contingent on environmental factors like pH, redox conditions, and the presence of other chemical species [3]. Arsenic and associated metals are naturally present in the Earth's crust, notably in geothermal discharges. Human-induced sources of As include mining activities, coal combustion, arsenic compounds in manufacturing processes, arsenical fungicides, insecticides, and herbicides in agriculture [4, 5].

Arsenic has been a subject of intense scientific scrutiny and concern due to its well-documented toxicological profile, encompassing carcinogenic, teratogenic, and mutagenic properties [6]. The contamination of surface soils by As and associated metals can impact organisms at the foundational levels of the coastal food web and affect agriculture [7]. More than 140 million people in over 70 nations are subject to acute and chronic exposure to exceeded As concentrations through ingestion, inhalation, and absorption via water and soil contact, leading to significant long-term health effects [8]. Acute As poisoning can result in gastrointestinal symptoms such as abdominal pain, vomiting, and diarrhea. Chronic exposure is linked to long-term health problems, including skin lesions, peripheral neuropathy, cardiovascular diseases, respiratory disorders, cancers, and hormonal imbalances [9]. Fe and Mn are essential for the human body and play a functional and structural role in biological systems [10]. Excessive Fe intake can lead to conditions like hemochromatosis, Fe toxicity, and gastrointestinal issues, potentially damaging organs such as the liver and heart [11]. Excessive Mn exposure, particularly in industrial settings, can cause manganism, a neurological disorder with symptoms similar to Parkinson's disease, as well as respiratory problems and liver/kidney damage [12]. Chronic Mn exposure can also affect cognitive function and mental health [13].

The recent economic transformation in Bangladesh has led to rapid industrialization and unplanned urbanization. Dumping untreated industrial waste, agrochemicals, poultry, and medical and household waste contaminates freshwater bodies [14]. Bangladesh is a land of rivers. Many rivers in Bangladesh carry large quantities of potentially toxic elements, which are finally deposited in the coastal regions through soil accretion [15]. Several studies have been carried out on the prevalence of As in groundwater, but fewer have focused on accreted soils [16, 17]. Surprisingly, there have been no studies on the occurrence and distribution of As in the coastal agricultural lands in Bangladesh. Manures, agrochemicals, compost amendments, and biosolids are the main ways potentially toxic elements contaminate agricultural soils [18, 19]. In addition to decreasing soil fertility and preventing seed germination [20] and causing a nutritional imbalance in plants [21], toxic substances in soil can stop soil microbial activity [22]. On the other hand, toxic heavy metal contamination can accumulate such elements in crops, and their subsequent appearance in the food chain can harm human health [1, 19]. Therefore, it is essential for coastal agricultural development and safe food production to determine the degree of contamination of potentially toxic elements in agricultural soils.

In the context of the lower Meghna River estuary, accretion significantly shapes the local landscape. Sediment deposition in this region, carried by the Meghna River, contributed to the gradual expansion of land areas along the coast [23]. This natural process affects the region's topography and hydrogeological dynamics, which may impact the mobility and distribution of elements like As, Fe, and Mn in soil and groundwater. The affected soil and groundwater form the basis of agricultural activity in the accreted lands of the Meghna River estuary. For heavy metal ions to be transferred from soil to plants, their roots are the most crucial point of interaction [24]. Arsenic, Fe, and Mn are prevalent metals in estuarine and coastal ecosystems, with significant implications for both environmental health and agricultural productivity. In the Meghna River estuary, the rapid deposition of sediment in the coastal accreted land has led to an accumulation of these metals, which can pose a potential risk to the surrounding communities through contaminated water, soil, and crops. Despite extensive studies on arsenic contamination in various regions, there is a lack of in-depth research that integrates the spatial distribution of these metals, their potential health risks, and their effects on agriculture in the Meghna River estuary. This study aims to fill this knowledge gap by mapping the spatial distribution of As, Fe, and Mn in the region, assessing the associated health risks, and evaluating the impact of metal contamination on agricultural sustainability. It offers a unique contribution by addressing the localized effects of these metals in an understudied region.

## 2. Materials and Methods

### 2.1. Study Area

Soil samples were collected from the accreted land along the greater Noakhali coast, situated between latitudes 22.4534°N and 23.1327°N and longitudes 90.8925°E and 91.3782°E. Noakhali, a dynamic coastal city near the Meghna River estuary in Southern Bangladesh, is part of the Ganges, Brahmaputra, and Meghna (GBM) Delta. The region features predominantly flat topography shaped by the tidal patterns of the Bay of Bengal, resulting in regular inundation of lowlands by tidal waters [25]. The area has experienced ongoing erosion and accretion processes, particularly influenced by the Meghna River's mouth. Sediments from the river are deposited here, contributing to a trend of land accretion extending southward in recent years. However, this natural process also leads to As and other metals accumulating in the soil, posing significant health hazards. The newly accreted land may act as a repository for As, Fe, and Mn, which could originate from underlying geological formations or be transported by river water. The fertile soils in Noakhali, enriched by silt deposits from the Meghna River estuary, support various agricultural activities. Major crops include rice (with three varieties: Aus, Boro, and Aman) as well as watermelon, peanuts, pulses, chilies, sugarcane, soybean, jute, mustard, potatoes, almonds, and various vegetables, especially in the winter and monsoon seasons [23]. Traditional farming practices such as manual harvesting and chemical and organic fertilizers are commonly employed.

### 2.2. Sample Collection and Processing

**Figure 1:**
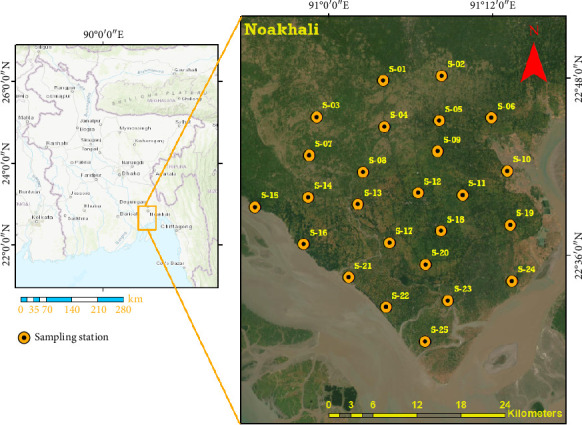
Geographical location of the study area and sampling sites of the lower Meghna River estuary, greater Noakhali coast.

A total of 25 soil samples were collected from 25 sampling stations on the lower Meghna River estuary (Figure 1). To cover the greater Noakhali district, sampling stations have been placed on both the east and west sides of the trunk route. Despite the limited communication infrastructure from the eastern to the western regions, the sampling stations were strategically positioned along the road network to ensure that the sampling design was sufficiently stretched to represent the entire area. The sample was collected in the post-monsoon season from September to October 2023. The samples were procured from the sedimentary accretion, which remains undisturbed. Surface soil samples (top 0–7 cm) were collected using a hand auger and well blended by a metal shovel. After collecting soil samples, a settling process was employed to eliminate excess water. Subsequently, each sample was individually transferred to porcelain dishes and transported to the laboratory. Samples were dried in an electric oven (Vinci Technologies SA-27 B, France) at approximately 40°C until a consistent weight was achieved. Then, the dried soil samples were homogenized, and 25 mm pellets were prepared through a pellet maker (Specac, UK) under 5 tons of pressure for subsequent elemental analysis via energy-dispersive X-ray fluorescence (EDXRF) (Epsilon-5, PANalytical, the Netherlands). EDXRF offers advantages such as nondestructive analysis, rapid sample preparation, and the ability to simultaneously analyze a wide range of elements. Unlike ICP-MS or OES, EDXRF is cost-effective, requires minimal sample preparation, and can be used for bulk and surface elemental analysis.

### 2.3. Elemental Analysis in Soil Sample Using EDXRF

EDXRF spectrometry (Epsilon-5, PANalytical, the Netherlands) was used to estimate the amounts of Mn, Fe, and As in soil samples. All the analyses were performed in triplicates for each sampling station. All sample pellets were exposed in a vacuum using a gadolinium (Gd) X-ray tube as the radiation source and a liquid nitrogen-cooled detector to monitor X-ray emissions. Calibration curves were constructed for As and associated Fe and Mn analysis of the soil samples using one standard reference material (marine sediment, IAEA 433). Each metal's calibration curve was created using the K and L X-ray intensities for the corresponding metals found in standard samples. The curves were further confirmed by plotting the atomic number of each metal against its sensitivity [26]. However, standard reference materials for quality control/quality assurance (QC/QA) were prepared and examined in the same manner as the experimental samples were prepared and analyzed. To ensure consistency between experiments, soil samples were analyzed alongside standard reference materials. To quantify the concentrations of each element, the system automatically analyzed the spectral data and calculated the net maximum intensities for each element. Relative errors were less than 5%, indicating high accuracy of the QC and QA systems. Standard reference materials have recovery rates between 94% and 106% (Table 1), demonstrating the robustness and reliability of the method [26].

### 2.4. Quantification of Soil Pollution

#### 2.4.1. Geo-Accumulation Index (Igeo)

Igeo is a useful tool for characterizing the contamination levels of sediments. Igeo value was estimated by the following equation:

(1) $$\begin{array}{c} {\text{Igeo}}_{n}=\log_{2}\left(\frac{C_{n}}{1.5\times Bn}\right), \end{array}$$

where *C*_*n*_ is the measured concentration of metal *n* in the sediment and *B*_*n*_ is the geochemical background value of the element in the background sample. In the current study, the elemental abundances of continental crust were used as the background data [27]. Factor 1.5 is introduced here to minimize the possible variations in the background values, which may be qualified for lithogenic effects. According to Müller [28], Igeo values were interpreted as Igeo ≤ 0 = uncontaminated, 0 ≤ Igeo ≤ 1  =  uncontaminated to moderately contaminated, 1 ≤ Igeo ≤ 2 = moderately contaminated, 2 ≤ Igeo ≤ 3 = moderately to heavily contaminated, 3 ≤ Igeo ≤ 4 = heavily contaminated, 4 ≤ Igeo ≤ 5 = heavily to extremely contaminated, and 5 ≤ Igeo = extremely contaminated.

#### 2.4.2. Enrichment Factor (EF)

For metal concentrations above uncontaminated background values, a normalized EF is often calculated to evaluate the anthropogenic influence on sediments using the following equation:

(2) $$\begin{array}{c} \text{EF}=\frac{\left(CX/CFe\right)\text{Sample}}{\left(CX/CFe\right)\text{Background}}. \end{array}$$

According to Taylor [29], metal contamination based on the EF value can be classified as follows.

EF < 1 = no enrichment, 1 < EF < 3 = minor enrichment, 3 < EF < 5 = moderate enrichment, 5 < EF < 10 = moderately severe enrichment, 10 < EF < 25 = severe enrichment, 25 < EF < 50 = very severe enrichment, and EF > 50 = extremely severe enrichment.

#### 2.4.3. Contamination Factor (CF)

The association between the background concentrations of each element and the reported soil concentrations of that element is known as the CF. CF is the ratio of the measured concentration to the natural abundance of a given metal:

(3) $$\begin{array}{c} \text{CF}=\frac{\left(\text{Metal Concentration}\right)\text{Sample}}{\left(\text{Metal Concentration}\right)\text{Background}}. \end{array}$$

The CF was categorized to track the degree of pollution caused by a specific metal over time. According to [30], CF was classified into the following four groups.

CF < 1 = low contamination, 1 ≤ CF < 3 = moderate contamination, 3 ≤ CF < 6 = considerable contamination, and CF ≥ 6 = very high contamination.

#### 2.4.4. Pollution Load Index (PLI)

The PLI is defined as the *n*th root of the product of CF of the *n* metals analyzed:

(4) $$\begin{array}{c} \text{PLI}={\left({\text{CF}}_{1}\times {\text{CF}}_{2}\times {\text{CF}}_{3}\times \cdots \cdots \cdots \cdots \times {\text{CF}}_{n}\right)}^{1/n}. \end{array}$$

Furthermore, it is classified by [31] as follows.

PLI = 0, indicates perfection; PLI = 1, baseline levels of pollutants are present; PLI > 1, progressive deterioration of estuarine quality.

#### 2.4.5. Potential Ecological Risk Index (PERI)

The PERI was initially suggested by Hakanson [30] and was used to evaluate the total potential ecological harm of heavy metals in soil. The potential ecological risk factor of a given metal (*E*_*r*_^*i*^) is defined as follows:

(5) $$\begin{array}{c} E_{r}^{i}=T_{r}^{i}\times \left(\frac{C_{i}}{C_{o}}\right). \end{array}$$

The following equation was used to calculate the PERI of sampling sites:

(6) $$\begin{array}{c} \text{PERI}=\overset{n}{\underset{i=0}{\sum }}T_{r}^{i}\times \frac{C_{i}}{C_{o}}, \end{array}$$

where *C*_*i*_ is the concentration of metal I in soil, *C*_*O*_ is the concentration of the same element in background soil, and *T*_*r*_^*i*^ is the biological toxicity factor of an individual element, which was determined for As = 10, Mn = 1, and Fe = 1 [30]. *E*_*r*_^*i*^ is the potential ecological risk factor of a single metal. PERI is the comprehensive potential ecological risk index of the metals. PERI values < 150, 150–300, 300–600, and > 600 are the evidence of low, moderate, considerable, and very high ecological risk, respectively.

#### 2.4.6. Nemerow Pollution Index (NPI)

NPI assesses the comprehensive pollution status of potentially toxic elements. Unlike other indices that measure average pollution levels, the NPI highlights the most critical pollutant by giving additional weight to the highest value among the parameters. The NPI index considers the average and the highest values of the single-factor pollution index, which can comprehensively reflect the average pollution degree of various pollutants in the soil and highlight the role of the most serious pollutants. The integrated NPI is expressed as follows:

(7) $$\begin{array}{c} P_{\text{ave}}=\frac{1}{n}\sum \frac{c_{i}}{s_{i}},\\ \text{NPI}=\sqrt{\frac{P_{\text{ave}}^{2}+P_{\max}^{2}}{2}}, \end{array}$$

where *P*_ave_ is the average value of the single pollution index for each heavy metal and *P*_max_ is the maximum value of the single pollution index for each heavy metal. The NPI is classified as follows: NPI ≤ 0.7, clean; 0.7 < NPI ≤ 1, warning limit; 1 < NPI ≤ 2, slight pollution; 2 < NPI ≤ 3, moderate pollution; and NPI > 3, heavy pollution.

### 2.5. Health Risk Assessment

#### 2.5.1. Noncarcinogenic Risk Assessment

In general, ingestion, dermal contact, and respiration are the three main routes to assessing the danger to human health. The ingestion pathways were only considered in this study. The exposure through these two pathways was determined using the following equations [32]:

(8) $$\begin{array}{c} {\text{ADD}}_{\text{ing}}=\frac{M\times \text{IR}\times \text{ER}\times \text{ED}}{\text{BW}\times \text{AT}},\\ {\text{ADD}}_{\text{derm}}=\frac{M\times \text{SA}\times \text{AF}\times \text{ABS}\times \text{ER}\times \text{ED}}{\text{BW}\times \text{AT}}\times 10^{-6}, \end{array}$$

where ADD_ing_ (mg/kg/day) and ADD_derm_ (mg/kg/day) are the average daily doses by ingestion and dermal absorption, respectively, for specific heavy metals; M represents the measured concentration of the metals in sediment; IR represents the ingestion rate (0.1 g/d for adults and 0.2 for children); ER is the exposure frequency (350 days/yr for both adults and children); ED represents the exposure duration (30 years for adults and 6 years for children); BW represents the body weight (70 kg for adults and 15 kg for children); AT represents the averaging time (10,950 days for adults and 2190 days for children); SA represents the exposed skin area (4350 cm^2^/event for adults and 1600 cm^2^/event for children); AF represents the sediment to skin adherence factor (0.7 mg/cm^2^ for adults and 0.2 mg/cm^2^ for children); and ABS is the dermal absorption factor (0.01 for adults and 0.001 for children) [33].

Based on ADD_ing_ and ADD_derm_ values, hazard quotients (HQs) were computed. The HQ is the ratio of ADD from ingestion and dermal exposure to their equivalent reference doses (RfD_ing_) and (RfD_derm_) [34]. HQ was computed using the following formula:

(9) $$\begin{array}{c} \text{HQ}=\frac{\text{ADD}}{RfD_{0}}. \end{array}$$

The R*fD*_0_ values of Fe, Mn, and As are 0.0003, 0.014, and 0.0003 mg/kg/day, respectively [33].

The hazard index (HI) values for ingestion and dermal pathways were determined.

(10) $$\begin{array}{c} \text{HI}=\sum \left({\text{HQ}}_{\text{ing}}+{\text{HQ}}_{\text{derm}}\right). \end{array}$$

#### 2.5.2. Carcinogenic Risk Assessment

The carcinogenic risk refers to the likelihood of an individual developing any cancer throughout their lifetime due to exposure to carcinogenic hazards. The individual excess lifetime cancer risk (IELCR) and cancer slope factor (SF) approach were used to estimate the carcinogenic risk. The carcinogenic risk was calculated for the ingestion route of exposure using the following equation:

(11) $$\begin{array}{c} \text{CR}=\text{ADD}\times \text{SF}, \end{array}$$

where ADD is the average daily dose value for ingestion and dermal contact pathways and SF is the slope factor for the heavy metal As = 1.5 [34]. The USEPA has not given SF values for Fe and Mn.

### 2.6. Statistical Analysis

All data were analyzed using PAST Version 4.17 and R (the R Foundation for Statistical Computing, Version 4.3.2). Pearson's correlation coefficient of the As and selected heavy metals in the soil was analyzed. The spatial distribution of As concentration was presented through color gradient maps constructed by inverse distance weighting (IDW) interpolation techniques in ArcGIS 10.8 software.

## 3. Results and Discussion

### 3.1. Abundance of As and Associated Metals (Fe and Mn)

**Figure 2:**
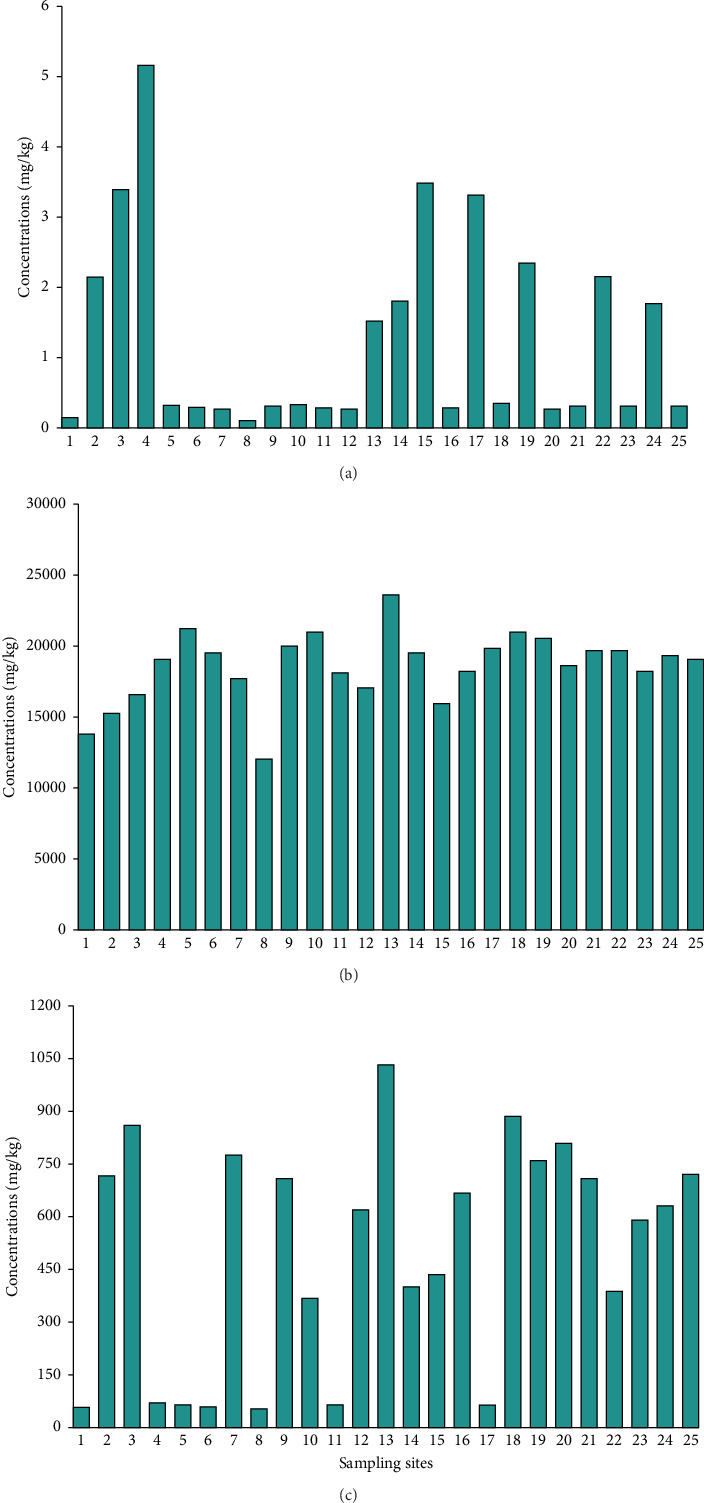
Mean concentration of (a) As, (b) Fe, and (c) Mn in the soil samples of the greater Noakhali coast, Noakhali, Bangladesh.

The mean concentration of metals followed the descending order: Fe (18,624 mg/kg) > Mn (482 mg/kg) > As (1.26 mg/kg). The results of this study align with the findings of previous research on the sediments of the lower Meghna River bank [25, 35], which found the same descending order of Fe, Mn, and As. A notable proportion of the stations, comprising around 70% of the total As (ranging from 0 to 0.50 mg/kg), indicated a prevalent but relatively low concentration of As in these areas (Figure 2(a)). The remaining stations exhibit higher concentrations, with the highest value recorded at station 4, where As reaches 5.16 mg/kg. In contrast, Fe concentrations vary significantly across the 25 stations, ranging from 12,000 mg/kg at station 8 to 810 mg/kg at station 13 (Figure 2(b)). Stations 5, 13, and 19 had the highest Fe concentrations, suggesting that localized factors contribute to the elevated levels. Manganese concentrations also show considerable variability, ranging from 50.60 mg/kg at station 8 to 1025.12 mg/kg at station 13 (Figure 2(c)). The wide range of Fe and Mn concentrations reflects the heterogeneous distribution of these metals in the coastal region, influenced by both natural factors such as geological conditions and sediment types, as well as anthropogenic factors like fertilizer use, irrigation, and industrial activities [36]. A comparison of As, Fe, and Mn concentrations in the coastal area soil samples of this study and the results reported worldwide, along with the different international standards, are presented in Table 2. Mainly, the concentration of As was 0.10–5.16 mg/kg in this study, much higher than that in the southern coastal soil of Bangladesh, ranging from 0 to 0.13 mg/kg [37]. The calmer coastal waters allow finer particles, such as those rich in iron oxides and clays, to settle out of the water column, resulting in higher Fe concentrations ranging from 12,000 to 23,810 mg/kg in the greater Noakhali coast compared to the southern coastal soils in Bangladesh reported as 890–47380 mg/kg [37]. Similarly, the Mn 50.60–1025 mg/kg concentration was higher than the southern coastal soils in Bangladesh, ranging from 92.50 to 199 mg/kg [37]. The present study found lower Fe concentrations at all sampling sites than the reference values of Rudnick and Gao for the upper continental crust [27], but at some sites, As and Mn concentrations were higher than those in the upper continental crust. Likewise, all stations showed lower shale values for As and Fe, although some sites showed higher values for Mn [51].

Globally, the concentrations of As, Fe, and Mn in this region are lower than those found in neighboring coastal areas such as India [38], Port Klang, Selangor, Malaysia [40], Peninsular Malaysia [41], the San Luis Potosí drainage basin, Mexico [43], the southeast coast of the Black Sea, Turkey [44], the Western Caspian Sea, Azerbaijan [48], and the coastline of the Erongo Region, Western Namibia [47] (Table 2). However, the concentrations of these metals in this study are higher than those reported for the coast of Dar es Salaam, Tanzania [45], and the Arabian Gulf, Saudi Arabia [39].

### 3.2. Spatial Distribution of Metals in Soil

**Figure 3:**
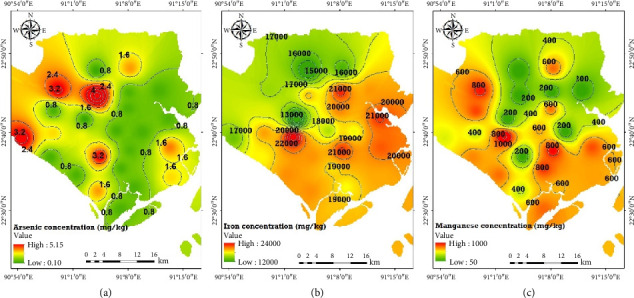
Spatial distribution of (a) As, (b) Fe, and (c) Mn concentration in the surface soil sample of the greater Noakhali coast using the inverse distance weighted technique (ArcGIS 10).

The spatial distribution of As in the greater Noakhali coastal region was mapped using the inverse distance weighted technique (IDWT) in ArcGIS 10, as shown in Figure 3. The highest concentrations of As were found in the west and northwest coastal regions near the Meghna River estuary, while lower concentrations were observed in the east, south, and northeast. However, higher As concentrations were also detected in the southwest and southeast regions. Iron concentrations were highest in the central coastal region, extending to the east, south, and southeast, with lower levels in the west and northwest. Manganese concentrations were moderate to high across the entire coastal belt, with the highest levels extending from the coast toward the central region and decreasing toward the north. The elevated As, Fe, and Mn levels in certain areas may result from natural geological processes, where rocks and minerals containing these metals erode and release into rivers and coastal areas. Industrial activities and certain pesticides and herbicides can also contribute to contamination by As and associated metals, particularly in regions near rivers or coastlines. In contrast, areas further from these sources, such as the central coast, show lower concentrations due to dilution by the ocean.

### 3.3. Assessment of Contamination Level

#### 3.3.1. Igeo

**Figure 4:**
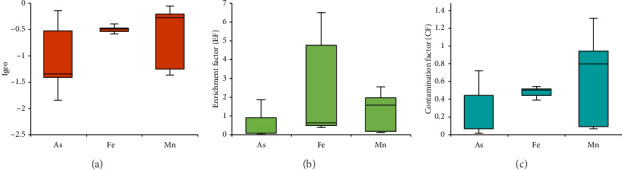
Geo-accumulation index (Igeo) (a), enrichment factor (EF) (b), and contamination factor (CF) (c) values of As, Fe, and Mn from the greater Noakhali coast region, Bangladesh. The bar shows mean values for each heavy metal with standard error.

The Igeo values of As, Fe, and Mn were estimated as −1.05, −0.50, and −0.55, respectively. The Igeo values for the analyzed metals were below zero for all the metals (Figure 4(a)), indicating that the greater Noakhali coast region is not contaminated by As, Fe, and Mn.

#### 3.3.2. EF

The mean EFs for the selected elements were calculated as shown in Figure 4(b). The values of Fe (2.08 ± 0.49) and Mn (1.33 ± 0.17) were over the enrichment level (EF < 1), while As (0.53 ± 0.12) showed the lowest mean of EF. EF values ranging from 0.50 to 1.50 typically mirror the inherent geological makeup of a given region. In contrast, EF values greater than 1.50 indicate the presence of external sources or human-induced weathering processes [52]. In this study, the EF value for As suggests that its presence is primarily due to crustal sources with minimal alteration. Meanwhile, Fe and Mn show moderate and minimal levels of alteration, respectively.

#### 3.3.3. CF and PLI

**Figure 5:**
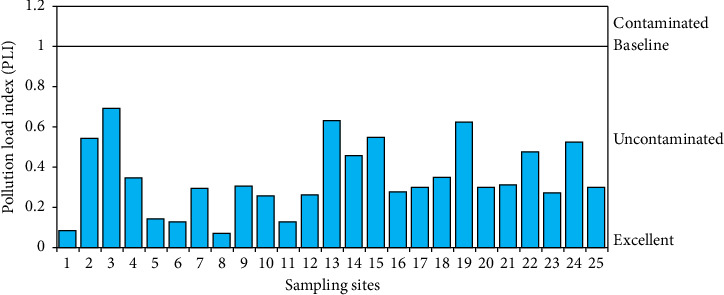
Pollution load index (PLI) values of As and associated heavy metals (Fe and Mn) for each sampling site on the greater Noakhali coast region, Bangladesh.

The mean CFs for the selected elements were calculated as shown in Figure 4(c). The CF values for As and other selected metals were less than 1, indicating a lower degree of contamination by As, Fe, and Mn [30]. The PLI values at the sampling sites ranged from 0.07 to 0.69, lower than the reference value of 1 (Figure 5). Therefore, it can be concluded that the soil is not polluted in terms of PLI value [31].

#### 3.3.4. PERI

**Figure 6:**
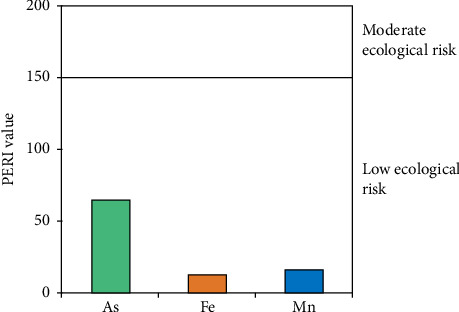
Potential ecological risk index (PERI) values of As, Fe, and Mn from the accreted land in the greater Noakhali coast, Bangladesh.

This study revealed that the potential ecological risk factor (Eri) for As, Fe, and Mn ranged from 0.21–10.75, 0.31–0.61, and 0.07–1.32 (Figure 6). The order of potential ecological risk factors of heavy metals in the soils of the Noakhali coastal region was As > Mn > Fe. The PERI values for As, Fe, and Mn were below 40, indicating a low ecological risk. PERI values for all sampling sites were well below 150, suggesting a low potential ecological risk for the soil samples collected from the coastal region of Noakhali. However, the gradual accumulation of these metals over time could still harm the environment, even without immediate damage. Arsenic can negatively impact plants and aquatic life by disrupting soil microorganisms and accumulating in crops, posing a threat to food security. Similarly, the slow buildup of Mn can harm soil biodiversity and alter environmental conditions, while Fe affects nutrient availability and water quality through runoff.

#### 3.3.5. NPI

The NPI values of the three potentially toxic elements in the lower Meghna River estuary of the greater Noakhali district reveal insights into the pollution levels in the region where Mn is the primary pollutant of concern. The NPI value of Mn was 1.13, indicating a slight pollution level (1 < NPI < 2), whereas the As and Fe values of NPI were less than 1, indicating low pollution. The NPI values for As and Fe for the study area were 0.80 and 0.65, respectively. Although the NPI value for As was less than 1, it indicated a warning limit for the toxic element (0.70 < NPI < 1). Although each pollutant (except Mn) may be within moderate limits, the combined loading of the three may have synergistic effects and further exacerbate their impacts on the ecosystem and human health, clearly highlighting the urgent need for integrated pollution management in the lower Meghna estuary.

### 3.4. Evaluation of Human Health Risk

#### 3.4.1. Noncarcinogenic Risk Assessment

In the exposure assessment phase, a specific method was used to evaluate human exposure to soil in the greater Noakhali coast, focusing on noncarcinogenic hazard exposure.  Table 3 presents the noncarcinogenic HQ for both ingestion and dermal intake, as well as the HI for the soil in this region. The HQ values for both exposure routes followed the order: Fe > Mn > As. The HI values for adults and children were below 1 for all metals, indicating that the metal content in the coastal soil is not expected to pose noncarcinogenic risks to human health through ingestion or dermal exposure [53]. While there is no immediate noncancer health risk from either ingestion or dermal contact, potential long-term effects must be considered. The gradual accumulation of these metals over time may lead to delayed health issues that may not be evident in short-term assessments. Arsenic, in particular, is of concern due to its association with various health problems from prolonged, low-level exposure, including skin lesions, cardiovascular diseases, developmental disorders in children, and even cancer [9]. However, at present, no immediate noncancer risk has been identified.

#### 3.4.2. Carcinogenic Risk Assessment

The estimated carcinogenic risk for As in the soil of the greater Noakhali coastal region for adults and children indicates that the carcinogenic risk from ingestion is higher for children than adults, whereas the risk from dermal exposure is lower for children (Table 3). Specifically, the ingestion cancer risk (CR_ing_) for children (2.39E − 05) and adults (2.56E − 06) slightly exceeds the acceptable range (1.00E − 06 to 1.00E − 04), suggesting a low carcinogenic effect. Carcinogenic risk refers to the likelihood of an individual developing cancer over their lifetime due to exposure to carcinogenic hazards. This study utilized the IELCR and cancer SF methods to assess the carcinogenic risk, specifically from ingestion, focusing on significant heavy metals [54]. Based on the dermal cancer risk (CR_derm_) values for both age groups, there appears to be no significant carcinogenic risk [55]. However, long-term ingestion of As can lead to cumulative effects and increase the risk of various cancers, including skin, lung, bladder, kidney, and liver cancer. While the immediate risk may seem low, prolonged exposure over many years or decades, especially in children who are more susceptible due to their smaller size, higher metabolism, and developing organs, can gradually result in significant health effects. The higher ingestion risk in children underscores their increased vulnerability to As-related health issues. Although dermal exposure is currently considered to have a lower carcinogenic risk, chronic skin contact with As can still lead to skin diseases like hyperkeratosis and lesions, potentially increasing the risk of skin cancer over time.

### 3.5. Correlation Among the Heavy Metals

**Figure 7:**
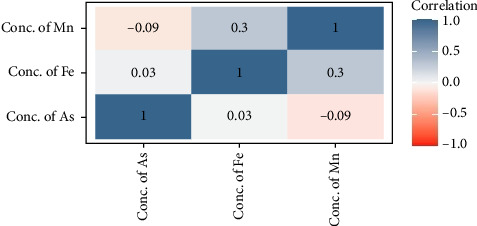
Pearson correlation coefficients (*r*) among As and associated heavy metals on the soil of the accreted land in the greater Noakhali coast region.

A Pearson correlation analysis was conducted on the metals in this study, revealing no significant relationships between As and other selected metals such as Fe and Mn. However, a weak positive correlation was found between Fe and Mn across the twenty-five sampling sites (coefficient of determination, *r*^2^ = 0.300; *p* < 0.05) (Figure 7). This positive correlation suggests that Fe and Mn may share a common source potentially either natural or anthropogenic. The presence of correlation among heavy metals can offer insights into their origin and migration [56]. In contrast, the absence of correlations between certain elements indicates that they are not influenced by a single factor [57].

### 3.6. Implication in Coastal Agriculture

Heavy metal contamination in agricultural soils is a widespread issue. While certain HMs, such as Fe and Mn, are essential for plant growth, their excessive accumulation can be harmful. High concentrations of HMs can disrupt plant functions, including ion homeostasis, photosynthesis, and enzyme activity [58]. According to the Ministry of the Environment, Finland, and the National Geochemical Survey of Australia (NGSA), the threshold values for As and Mn in soils are 5–20 mg/kg and 500–1000 mg/kg, respectively [59]. Although the As levels in this study fall within the lower range, its high toxicity remains a concern, as it can hinder plant development and contaminate groundwater used for irrigation, potentially posing a health risk to local populations [60]. Arsenic, along with associated metals like Fe and Mn, can negatively impact plants by damaging the fine structure of chloroplasts, disrupting chlorophyll a/b ratios, impairing the biosynthesis of photosynthetic machinery, and altering pigment composition in grana and stroma membranes.

Furthermore, these metals can enter the food chain and harm human health [58]. Iron toxicity, particularly from concentrations exceeding 10,000 mg/kg in this study, can reduce crop yields, hinder nutrient uptake, and degrade soil quality. Similarly, elevated Mn levels, especially at the high end of the range, can cause plant toxicity symptoms such as stunted growth and leaf chlorosis [58].

Long-term exposure to high concentrations of heavy metals like As, Fe, and Mn can harm soil quality, reducing plant fertility and nutrient availability. Elevated Fe and Mn levels can interfere with the uptake of essential nutrients like calcium, magnesium, and phosphorus, causing crop deficiencies. This results in gradual declines in agricultural production. Collaborative efforts among biologists, soil scientists, and farmers can develop site-specific management strategies for the sustainable use and safe disposal of metal-contaminated soils, ensuring long-term agricultural productivity. Managing agricultural soils contaminated with As, Fe, and Mn requires regular soil testing, pH adjustment, and soil amendments like lime, organic matter, and phosphorus to reduce metal bioavailability. Phytoremediation using tolerant plants can help remove contaminants, while crop selection and rotation minimize uptake. Soil washing and microbial remediation can further address contamination. Proper irrigation and drainage, balanced fertilization, and chelating agents are also important. Compliance with regulatory standards, best management practices, and community education are essential for sustainable and safe agricultural practices in contaminated areas.

## 4. Conclusions

This study found elevated As, Fe, and Mn levels in several coastal regions of the Meghna River estuary. Despite this, assessments showed that the soil is uncontaminated with these metals and poses a low ecological risk. The HI indicated no noncarcinogenic health risks from As exposure through ingestion or dermal contact, with low cancer risk for both adults and children. However, elevated As concentrations in specific stations could harm coastal agriculture and pose risks through crop accumulation.

The present study could help identify As, Fe, and Mn contamination in agricultural soils and assess toxicity risks. It guides soil fertility management by optimizing fertilizer application, improving nutrient uptake, and preventing deficiencies. Soil testing also supports sustainable agricultural practices, informs crop selection, and helps develop remediation strategies to reduce contamination. Overall, it enhances scientific understanding of metal dynamics in soils and aids in developing strategies for sustainable, productive, and safe agricultural systems. To obtain a comprehensive picture of the contamination of soil by metals in the coastal region of Noakhali, future research should take into account (i) additional potentially toxic elements, (ii) the vertical distribution of these elements, and (iii) seasonal variations of accumulation of these elements.

## Figures and Tables

**Table 1:** The method detection limit (mg/kg) for EDXRF and comparative values of the measured and certified concentrations of standard reference materials (marine sediment, IAEA 433).

Elements	Method detection limit (mg/kg)	Measured conc. (mg/kg)		Certified conc. (mg/L)	RE (%)	CV (%)
		Mean	SD			
Mn	0.74	1125	64.40	1180	4.70	5.72
Fe	0.29	26,112	738	27,100	3.63	2.83
As	0.35	17.70	0.64	18.30	3.25	3.25

**Table 2:** Comparison of total As, Fe, and Mn in the surface soil of the present study and other studies in Bangladesh and worldwide.

Locations	Descriptive statistical parameters	Total As concentrations (mg/kg)	Total Fe concentrations (mg/kg)	Total Mn concentrations (mg/kg)	References
Greater Noakhali coast, Bangladesh	Range (mean)	0.10–5.16 (1.26)	12,000–23,810 (18,677.20)	50.60–1025.12 (497.58)	Present study
Southern coastal sediments in Bangladesh	Range	0–0.13	890–47380	92.48–198.63	Sultana et al. [37]
Sediments of the Bay of Bengal coast	Range	NA	895–1456	3.91–6.97	Khan et al. [14]
Major district, India	Range (mean)	0.20–197 (26.83)	4.25–2,31,244 (28,185.65)	0.10–24,365 (1668.97)	Kumar et al. [38]
Arabian Gulf, Saudi Arabia	Mean	1.606	7552	113.97	Alharbi and El-Sorogy [39]
Port Klang, Selangor, Malaysia	Mean ± SD	475.26 ± 47.32	814 ± 22	1838.93 ± 102.34	Tavakoly et al. [40]
Peninsular, Malaysia	Range	21.81–59.49	12,973–48916	NA	Cheng and Yap [41]
Doha, east coast of Qatar	Mean	2.56	1972	28.2	Hasna et al. [42]
San Luis Potosí drainage basin, Mexico	Range	39–1500	19,025–94,639	443–2499	Montes-Avila et al. [43]
Southeast coast of the Black Sea, Turkey	Mean	7.36	32,582.40	571.87	Kodat and Tepe [44]
Dar es Salaam coast, Tanzania	Range	0.20–1.30	461–5352	17–219	Rumisha et al. [45]
Songkhla lagoon, Thailand	Mean	5.64	NA	176.74	Pradit et al. [46]
Coastline of Erongo Region, Western Namibia	Range	2.10–6.10	1587–8221	22.0–86.0	Onjefu, Kgabi, and Taole [47]
Western Caspian Sea, Azerbaijan		13	47,200	850	Ahmadov et al. [48]
Asturian Coast, Spain	Range	3.50–55.30	1700–3860	NA	Sanz-Prada et al. [49]
Huautla district, Mexico	Mean	4.49	NA	627	Barats et al. [50]
Upper continental crust (UCC)		4.80	39,200	775	Rudnick and Gao [27]
Average shale value (ASV)		13	47,200	850	Turekian and Wedepohl [51]

**Table 3:** Hazard quotients (HQs), hazard index (HI), and carcinogenic risk (CR) values for As, Fe, and Mn in the greater Noakhali coastal soils.

	Noncarcinogenic risks for child			Noncarcinogenic risks for adult			Carcinogenic risk for child		Carcinogenic risk for adult	
	HQ_ing_	HQ_derm_	HI	HQ_ing_	HQ_derm_	HI	CR_ing_	CR_derm_	CR_ing_	CR_derm_
As	5.31E − 02	8.50E − 05	5.32E − 02	5.69E − 03	1.73E − 03	7.42E − 03	2.39E − 05	3.82E − 08	2.56E − 06	7.80E − 07
Fe	3.41E − 01	5.46E − 04	3.42E − 01	3.66E − 02	1.11E − 02	4.77E − 02				
Mn	4.54E − 02	7.27E − 05	4.55E − 02	4.87E − 03	1.48E − 03	6.35E − 03				

## Data Availability

The data that support the findings of this study are available on request from the corresponding author. The data are not publicly available due to privacy or ethical restrictions.
